# Integrating deep learning in public health: a novel approach to PICC-RVT risk assessment

**DOI:** 10.3389/fpubh.2024.1445425

**Published:** 2025-01-07

**Authors:** Yue Li, Shengxiao Nie, Lei Wang, Dongsheng Li, Shengmiao Ma, Ting Li, Hong Sun

**Affiliations:** ^1^Institute of Biomedical Engineering, Chinese Academy of Medical Sciences and Peking Union Medical College, Beijing, China; ^2^School of Electronic and Information Engineering, TianGong University, Tianjin, China; ^3^Department of Nursing, Beijing Hospital, National Center of Gerontology, Institute of Geriatric Medicine, Chinese Academy of Medical Sciences, Beijing, China; ^4^School of Nursing, Chinese Academy of Medical Sciences and Peking Union Medical College, Beijing, China; ^5^Beijing Hospital, National Center of Gerontology, Institute of Geriatric Medicine, Chinese Academy of Medical Sciences, Beijing, China

**Keywords:** artificial intelligence, machine learning, peripherally inserted central catheter, time-to-event, thrombosis

## Abstract

**Background:**

Machine learning is pivotal for predicting Peripherally Inserted Central Catheter-related venous thrombosis (PICC-RVT) risk, facilitating early diagnosis and proactive treatment. Existing models often assess PICC-RVT risk as static and discrete outcomes, which may limit their practical application.

**Objectives:**

This study aims to evaluate the effectiveness of seven diverse machine learning algorithms, including three deep learning and four traditional machine learning models, that incorporate time-series data to assess PICC-RVT risk. It also seeks to identify key predictive factors for PICC-RVT using these models.

**Methods:**

We conducted a retrospective multi-center cohort study involving 5,272 patients who underwent PICC placement. After preprocessing patient data, the models were trained. Demographic, clinical pathology, and treatment data were analyzed to identify predictive factors. A variable analysis was then conducted to determine the most significant predictors of PICC-RVT. Model performance was evaluated using the Concordance Index (c-index) and the composite Brier score, and the Intraclass Correlation Coefficient (ICC) from cross-validation folds assessed model stability.

**Results:**

Deep learning models generally outperformed traditional machine learning models in terms of predictive accuracy (mean c-index: 0.949 vs. 0.732; mean integrated Brier score: 0.046 vs. 0.093). Specifically, the DeepSurv model demonstrated exceptional precision in risk assessment (c-index: 0.95). Stability varied with the number of predictive factors, with Cox-Time showing the highest ICC (0.974) with 16 predictive factors, and DeepSurv the most stable with 26 predictive factors (ICC: 0.983). Key predictors across models included albumin levels, prefill sealant type, and activated partial thromboplastin time.

**Conclusion:**

Machine learning models that incorporate time-to-event data can effectively predict PICC-RVT risk. The DeepSurv model, in particular, shows excellent discriminative and calibration capabilities. Albumin levels, type of prefill sealant, and activated partial thromboplastin time are critical indicators for identifying and managing high-risk PICC-RVT patients.

## 1 Introduction

In modern medical settings, the Peripherally Inserted Central Catheter (PICC) has become an indispensable tool for administering long-term intravenous therapies such as cancer treatments, prolonged antibiotic courses, and nutritional support, due to its significant advantages (1, 2). Despite its numerous benefits, the use of PICC is associated with certain risks, primarily the development of PICC-related venous thrombosis (PICC-RVT). The incidence of PICC-RVT in critically ill patients varies widely, from 13 to 91% (3), and about 30% of these cases can lead to more severe complications such as pulmonary embolism, post-thrombotic syndrome, and limb paralysis, which, in extreme cases, could result in death (4–7).

Given these risks, there is a crucial public health interest in developing effective prediction and management strategies for PICC-RVT. This study leverages machine learning algorithms, which represent an advancement over traditional statistical methods like regression, in predicting PICC-RVT outcomes, thus enhancing therapeutic effectiveness and reducing complications. Previous research has demonstrated that machine learning models offer more robust discriminative accuracy (8–13). However, the direct applicability of these models in routine clinical practice remains limited due to several factors, including the lack of integration of time-to-event data and the absence of comprehensive model validation metrics like the Brier score (8, 14, 15).

Our study proposes a novel approach by incorporating event occurrence time data and extensive risk factor categorization within machine learning frameworks to predict overall survival rates of PICC-RVT across large datasets. We evaluated three neural network extension algorithms and four ensemble learning algorithms on their ability to predict these outcomes in varied patient cohorts. A detailed feature analysis helped us refine the models and stratify PICC-RVT patients into distinct risk categories, thereby facilitating more precise clinical decision-making.

This research not only contributes to clinical practice by providing tools for better risk management of PICC-RVT but also aligns with public health goals by potentially informing policy decisions related to the safe use of PICC lines. By reducing the incidence of severe complications, our findings could lead to improved patient outcomes and reduced healthcare costs, emphasizing the relevance of integrating advanced predictive technologies in public health strategies.

## 2 Materials and methods

### 2.1 Data collection and ethical considerations

In a comprehensive retrospective multi-center cohort study conducted from May 2015 to July 2023, we examined patients receiving PICC, with data collection spanning 27 hospitals in China, as outlined in Table 1. This study was authorized by the China Clinical Trials Registry (Registration Number: ChiCTR2300070265).

**Table 1:** Comparison of no complication and complication set demographic information.

Variable	No-PICC-RVT (*N* = 2,928)	PICC-RVT (*N* = 525)
Age (years)	52.62 ± 11.43	55.92 ± 11.21
Height (cm)	162.35 ± 8.04	164.89 ± 8.34
Weight (kg)	62.74 ± 11.26	64.17 ± 12.23
White blood cell (10×10^9^/L)	6.85 ± 14.60	6.30 ± 3.41
Hemoglobin (g/L)	126.60 ± 33.20	128.22 ± 38.38
Platelet (10^9^/L)	246.87 ± 86.28	235.83 ± 85.88
D-Dimer (mg/L)	1.10 ± 4.03	1.26 ± 4.63
Prothrombin time (s)	13.13 ± 2.74	12.80 ± 2.58
Fibrinogen (g/L)	3.49 ± 1.26	3.63 ± 1.32
Activated partial prothrombin time (s)	29.46 ± 11.51	27.54 ± 5.08
Albumin (g/L)	33.10 ± 17.07	38.86 ± 9.07
C-reactive protein (mg/L)	11.72 ± 25.87	12.55 ± 23.36
Catheter vessel diameter ratio	0.33 ± 0.07	0.34 ± 0.07
Gender (Male/Female):	897 (30.6%)/2,031 (69.4%)	233 (44.3%)/293 (55.7%)
Education		
No record/Primary school	1,837 (62.67%)/589 (20.10%)	397 (75.61%)/76 (14.47%)
Middle and high school/Bachelor degree or above	304 (10.37%)/201 (6.85%)	37 (7.04%)/15 (2.85%)
Tumor staging		
I/II	268 (9.15%)/583 (19.91%)	58 (11.04%)/61 (11.62%)
III/IV	892 (30.46%)/1,185 (40.47%)	177 (33.71%)/228 (43.43%)
Chemotherapy cycle		
No chemotherapy/1–4	1,006 (34.35%)/1,076 (36.74%)	95 (18.09%)/287 (54.66%)
5–8/>8	737 (25.17%)/109 (3.72%)	129 (24.57%)/14 (2.66%)
Main diagnosis of admission		
Respiratory system	476 (16.2%)	102 (19.4%)
Circulatory system	14 (0.4%)	3 (0.05%)
Digestive system	626 (21.3%)	155 (29.52%)
Motion system	14 (0.4%)	1 (0.1%)
Nervous system	19 (0.6%)	3 (0.5%)
Urinary system	20 (0.6%)	2 (0.3%)
Reproductive system	179 (6.1%)	37 (0.7%)
Mammary glands	1,287 (43.9%)	159 (30.2%)
Blood system	150 (5.1%)	28 (5.3%)
Ear, nose, throat, head, neck and mouth	101 (3.4%)	24 (5.1%)
Others	42 (1.4%)	11 (2.0%)
Reason for catheter retention		
Chemotherapy drugs	2,392 (81.69%)	460 (87.62%)
Hypertonic fluid	189 (6.45%)	36 (6.86%)
Long-term infusion	146 (4.99%)	25 (4.76%)
Others	7 (0.23%)	1 (0.19%)
Puncture blood vessel		
Basilic vein	2,682 (91.60%)	492 (93.71%)
Brachial vein	173 (5.91%)	23 (4.38%)
Cephalic vein	49 (1.67%)	3 (0.57%)
Others	24 (0.82%)	7 (1.33%)
Conduit mode		
Bard PICC Catheter 4F	2,898 (98.97%)	523 (99.7%)
Others	30 (1.03%)	2 (0.3%)
Type of PICC		
Tri-valve PICC	2,789 (95.25%)	443 (82.48%)
Front opening	73 (2.49%)	55 (10.48%)
Others	67(2.25%)	37(7.05%)
Position of catheter tip		
T7 and Lower than T7	1,348 (46.06%)	230 (43.9%)
Upper than T6	1,580 (53.96%)	295 (56.1%)
PICC catheterization method		
Under ultrasound guidance	2,593 (88.56%)	496 (94.48%)
Ultrasound Guidance Combined with ECG	223 (7.62%)	13 (2.48%)
Others	112 (3.9%)	16 (3.05%)
Puncture complications		
None	2,769 (94.57%)	515 (98.10%)
Malposition	137 (4.68%)	9 (1.71%)
Others	22 (0.75%)	1 (0.19%)
Positioning method of catheter tip		
X-ray	2,099 (71.69%)	498 (94.86%)
ECG + X ray	252 (8.61%)	11 (3.05%)
Others	577 (19.71%)	16 (2.1%)
Type of disinfectant		
Dextrose Chlorhexidine alcohol Solution	582 (19.88%)	107 (20.38%)
Ethanol + Iodine Tincture	2,340 (79.92%)	418 (79.62%)
Others	6 (0.2%)	0 (0%)
Connector Type		
Positive pressure connector	2,538 (86.68%)	388 (73.90%)
Heparin cap	340 (11.61%)	123 (23.43%)
Negative pressure connection	27 (0.92%)	7 (1.33%)
Balance pressure connector	2 (0.07%)	4 (0.76%)
StatLock fixed PICC (No/Yes)	2,677 (91.4%)/251 (8.6%)	462 (88%)/63 (12%)
Pre-filled sealing fluid (No/Yes)	1,988 (67.90%)/940 (32.10%)	488 (85.33%)/77 (14.67%)
Type of sealing fluid (Normal/Heparin)	2,877 (98.26%)/51 (1.74%)	508 (96.76%)/17 (3.27%)
Catheter lumen number (Single/Others)	2,923 (99.83%)/5 (0.17%)	525 (100%)/0 (0%)
Puncture site (Left/Right)	1,465 (50.03%)/1,463 (49.97%)	284 (54.10%)/241(45.90%)
Number of punctures (1/>1)	2,801 (95.66%)/124 (4.34%)	513 (97.71%)/12 (2.29%)
History of Hypertension (No/Yes)	2,386 (81.49%)/542 (18.51%)	446 (84.95%)/79 (15.05%)
History of Diabetes (No/Yes)	2,687 (91.77%)/241 (8.23%)	488 (92.95%)/37 (7.05%)
History of coronary heart disease (No/Yes)	2,897 (98.94%)/31 (1.06%)	523 (99.62%)/2 (0.38%)
Past deep vein thrombosis and thrombophlebitis (No/Yes)	2,900 (99.04%)/28 (0.96%)	515 (98.10%)/10 (1.90%)
History of deep vein catheterization (No/Yes)	2,754 (94.06%)/174 (5.94%)	480(91.43%)/45(8.57%)
Platinum drugs (No/Yes)	1,903 (64.99%)/1,025 (35.01%)	245(46.67%)/280(53.33%)
Anthracycline drugs (No/Yes)	1,925 (65.74%)/1,003 (34.26%)	436 (83.05%)/89 (16.95%)
Alkylating agents (No/Yes)	1,850 (63.18%)/1,078 (36.82%)	425 (80.95%)/100 (19.05%)
immune modulators (No/Yes)	2,926(99.93%)/2(0.07%)	52 5(100%)/0 (0%)
Targeted drugs (No/Yes)	2,424 (82.79%)/502 (17.21%)	450 (85.71%)/75 (14.3%)
Plant alkaloids (No/Yes)	1,562 (53.35%)/1,366 (46.65%)	335 (63.81%)/190 (36.19%)
Anti-metabolite drugs (No/Yes)	2,323 (79.34%)/605 (20.66%)	339 (76.0%)/126 (24.0%)
Corticosteroid medications (No/Yes)	2,887 (98.6%)/41 (1.40%)	519 (98.86%)/6 (1.14%)
Topoisomerase Drugs (No/Yes)	2,653 (90.61%)/275 (9.39%)	451 (85.9%)/74 (14.1%)
Other chemotherapy drugs (No/Yes)	2,462 (84.08%)/466 (15.92%)	452 (86.1%)/73 (13.9%)

PICC insertions were executed by certified nurses with specialization in intravenous therapy, who were accredited by provincial or national nursing associations in China. These nurses received extensive training on PICC-related knowledge and diagnostic standards to ensure the standardization and precision of the procedure before the study began. The accurate placement of the catheter tip was confirmed by professional physicians, while maintenance care, including the changing of the transparent dressing (with the initial change within 24 h post-insertion), was conducted weekly by trained nurses.

This study employed color Doppler ultrasound to monitor thrombotic complications. Clinically, thrombotic complications post-PICC placement are identified if a patient exhibits symptoms such as pain, swelling, erythema, increased skin temperature, or venous distension in the limb, shoulder, neck, or chest on the catheter side, accompanied by one or more of the following ultrasound findings: (a) inability to compress the venous lumen, (b) filling defects within the lumen’s blood flow signal, (c) significant echogenicity within the lumen, (d) loss of phasic changes in venous flow spectrum, (e) weakened or absent Vasalva response, and (f) reduced or absent blood flow augmentation upon distal limb compressiont (14).

From the study’s outset, we collected data on demographic characteristics, medical histories, and previous experiences with deep venous catheterization and venous thromboembolism from medical records and participant reports. At the initial assessment, clinical and physical examination results were documented, along with laboratory indicators like white blood cell count, hemoglobin, and platelet count. After catheter insertion, detailed information regarding the catheter and puncture-associated data was continually collected. Treatment-related characteristics, including the patient’s activity status and ultrasound findings, were updated weekly.

### 2.2 Data preparation

#### 2.2.1 Data cleaning

The inclusion criteria for patients are: (a) aged over 18; (b) requiring intravenous administration of antineoplastic drugs or nutritional support; (c) a life expectancy of more than 30 days post-PICC insertion. The exclusion criteria are incomplete relevant data or record data anomaly.

#### 2.2.2 Data transformation and feature engineering

During the data collection phase of this study, several predictive features were initially categorized as categorical variables or Boolean values. These data underwent transformation to meet the model’s requirements. Specifically, one-hot encoding was utilized for variables such as the type of chemotherapy drugs. For instance, if the types of chemotherapy drugs are “Drug A,” “Drug B,” and “Drug C,” the one-hot encoding process transformed these categories into binary vectors as follows:


$$\begin{array}{l} \text{Drug }A:\left[1,0,0\right]\\ \text{Drug }B:\left[0,1,0\right]\\ \text{Drug }C:\left[0,0,1\right] \end{array}$$

This transformation converts categorical variables into a binary format, facilitating their processing by machine learning algorithms.

For continuous variables like albumin levels, feature scaling was applied to normalize the measurement scales of various features. The normalization process can be represented by the following formula:


$$X^{\prime }=\frac{X-\mu }{\sigma }$$

where X is the original value, *μ* is the mean of the dataset, and *σ* is the standard deviation. This standardization ensures that the albumin levels have a mean of 0 and a standard deviation of 1, enhancing algorithm performance and expediting model convergence. Additionally, feature scaling improves the model’s interpretability, making its outputs more straightforward for clinical physicians to comprehend and utilize.

#### 2.2.3 Feature selection

The study performed univariate analyses on selected data to investigate the correlation between categorical variables and thrombosis after PICC catheterization. Logistic regression models were employed to assess the relationship between both categorical and continuous variables with the incidence of thrombotic complications. Statistical analyses encompassed the calculation of Odds Ratios, 95% confidence intervals, and *p*-values, with a *p*-value threshold of less than 0.05 set for determining statistical significance. Factors significantly predicting thrombotic complications post-PICC catheterization were pinpointed and then integrated into the model as predictive features to forecast the likelihood of event occurrence.

#### 2.2.4 Learning algorithms

Three neural network-based algorithms—time-dependent Cox model (Cox-Time), DeepSurv, and DeepHit (16–18)—and four machine learning-based algorithms—Random Survival Forest (RSF), Mortality Prediction using Logistic Regression (MP-LogitR), Mortality Prediction using AdaBoost (MP-AdaBoost), and Threshold Regression (ThresReg) (19–22)—were selected for training. These algorithms, which have been utilized in medical survival analysis, were chosen for their reported performance in existing literature.

##### 2.2.4.1 DeepHit

DeepHit, a deep learning-based discrete-time survival analysis model, is specifically engineered to handle competing risks. This model utilizes a probability mass function estimation, which is achieved through a shared sub-network alongside multiple cause-specific sub-networks, thereby facilitating the discretization of continuous-time data. DeepHit’s architectural framework provides exceptional flexibility and accuracy in predicting survival time, representing a notable advancement in survival analysis (16, 23).

##### 2.2.4.2 DeepSurv

DeepSurv is a survival analysis model that leverages deep neural networks to enhance the Cox proportional hazards framework. It excels in capturing the intricacies of survival data through a nonlinear risk function, thereby facilitating personalized risk assessments across diverse clinical and biomedical datasets. By combining contemporary deep learning approaches with classical survival analysis methodologies, DeepSurv makes it possible to forecast outcomes without the need for specifying interaction terms, setting it apart from traditional models that necessitate such adjustments (17, 24).

##### 2.2.4.3 Cox-time

Cox-Time is a neural network-based survival analysis model designed to circumvent the proportional hazard assumption of traditional Cox models. It can process continuous-time data and uses time-dependent covariates to effectively model non-proportional hazards variables, similar to the approach in the DeepSurv algorithm. Cox-Time focuses on providing precise predictions of survival time, especially when dealing with data that exhibit complex relationships and time-dependent characteristics (16).

##### 2.2.4.4 Random survival forest

RSF is a powerful non-parametric survival analysis technique that combines multiple decision trees to handle survival time data (25). This method automatically addresses complex interactions and nonlinear relationships between variables, providing precise estimates of individual survival time distributions. RSF is particularly well-suited for high-dimensional data environments, capable of effectively identifying and evaluating significant predictive factors, thus playing a crucial role in the field of survival analysis (19).

##### 2.2.4.5 MP-LogitR

MP-LogitR is a survival analysis method that integrates machine learning techniques with survival data (20). It updates the dataset at different time intervals and uses traditional machine learning algorithms such as logistic regression to predict the survival status of patients, thereby assessing the survival risk at various time points. This method trains classifiers independently for each time point, offering a dynamic and precise risk assessment tool suitable for continuous-time survival analysis (26).

##### 2.2.4.6 MP-AdaBoost

MP-AdaBoost is a machine learning-based survival model designed for analyzing survival time data (21). This model employs the AdaBoost algorithm to predict the survival status of patients at various time intervals and, based on this, assesses the risk score for patients at each time point. MP-AdaBoost independently trains classifiers for each time interval, providing AdaBoost-based survival predictions for each time point, thereby offering a dynamic and detailed risk assessment for survival analysis (27).

##### 2.2.4.7 ThresReg

ThresReg is a threshold regression model used in survival analysis, particularly effective in scenarios where thresholds or boundaries exist (22). ThresReg models data by identifying key thresholds that impact survival time, allowing for dynamic assessment of the risk associated with survival events. This method is especially suitable for survival time prediction in datasets where clear demarcation points or thresholds are present (28).

#### 2.2.5 Model training and validation

After preprocessing, the data were utilized to train machine learning algorithms that account for event occurrence times, focusing on disease-specific and overall survival rates as outcomes. The training regimen for traditional machine learning algorithms such as RSF, MP-LogitR, MP-AdaBoost, and ThresReg was rigorously developed and executed. These algorithms applied five-fold cross-validation, dividing the dataset into 80% for training and 20% for testing. Notably, for the RSF model, bootstrapping without replacement was implemented to construct 1,000 independent decision trees.

For deep learning models like DeepSurv, Cox-Time, and DeepHit, Python 3.8 was used for development, with the Adam optimizer and an initial learning rate of 0.001. Hyperparameters including hidden layers, nodes per layer, dropout, and batch size were fine-tuned using five-fold cross-validation. Selection was based on the analysis of training and validation learning curves, manually calculated to identify the highest concordance index and lowest Brier score, ensuring an optimal model fit.

#### 2.2.6 Model performance evaluation

##### 2.2.6.1 Concordance index (C-index)

The C-index is an essential statistical tool for evaluating the performance of prediction models, particularly in survival analysis and time-to-event data. It measures the model’s accuracy in predicting the sequence of event occurrences, indicating the proportion of paired samples whose occurrence order is correctly predicted by the model (29). Mathematically, the C-index is defined as:


$$C-\text{index}=\frac{1}{N}\sum_{i<j}I\left(T_{i}<T_{j}\right)I\left({\overset{⌢}{T}}_{i}<{\overset{⌢}{T}}_{j}\right)+I\left(T_{i}=T_{j}\right)I\left({\overset{⌢}{T}}_{i}={\overset{⌢}{T}}_{j}\right)$$

where I is the indicator function, $T_{i}$ and $T_{j}$ are the true survival times, and $T_{i}$ and $T_{j}$ are the predicted survival times. Values range from 0.5 (indicating no better accuracy than random guessing) to 1.0 (denoting perfect prediction accuracy). In survival analysis, it assesses whether the model accurately identifies individuals at varying risk levels, with higher C-index values signifying superior predictive ability. The mean C-index reported in our study is the average C-index obtained from multiple cross-validation folds, providing a robust estimate of the model’s discriminatory power (30, 31).

##### 2.2.6.2 Brier scores (B-score)

The B-Score, established by Glenn W. Brier in 1950, serves as a critical measure for evaluating the accuracy of probability predictions, particularly in binary scenarios. It quantifies prediction accuracy by calculating the mean squared difference between the predicted probabilities and actual outcomes. For survival models, the integrated Brier Score is used to evaluate the performance of the model over time (15). The integrated Brier Score is given by:


$$B-\text{Score}=\frac{1}{\tau }\int_{0}^{\tau }\frac{1}{N}\sum_{i=1}^{N}{\left(\overset{⌢}{S}\left(t\left|X_{i}\right.\right)-I\left(T_{i}\leq t\right)\right)}^{2}dt$$

where $\overset{⌢}{S}\left(t\left|X_{i}\right.\right)$ is the predicted survival probability at time t for subject $i_{t}$, $I\left(T_{i}\leq t\right)$ is an indicator of whether the event has occurred by time t, and τ is the maximum follow-up time. The Brier score ranges from 0 (indicating perfect prediction) to 1 (indicating the poorest prediction), offering a clear, quantitative metric for assessing model performance. The mean integrated Brier Score is the average score across different time points and cross-validation folds, providing a comprehensive measure of the model’s calibration and accuracy.

##### 2.2.6.3 Model stability

Model stability evaluates the robustness of machine learning models against variations in training data, reflecting on the model’s generalizability and reliability. Following a method akin to Turney’s approach (32), this study assesses stability using five-fold cross-validation and measures it by the intraclass correlation coefficient (ICC) of performance metrics across different test subsets. Model performance is further evaluated using the average and standard deviation of the C-index across folds and the combined Brier Score.

## 3 Results

### 3.1 Population characteristics

**Figure 1:**
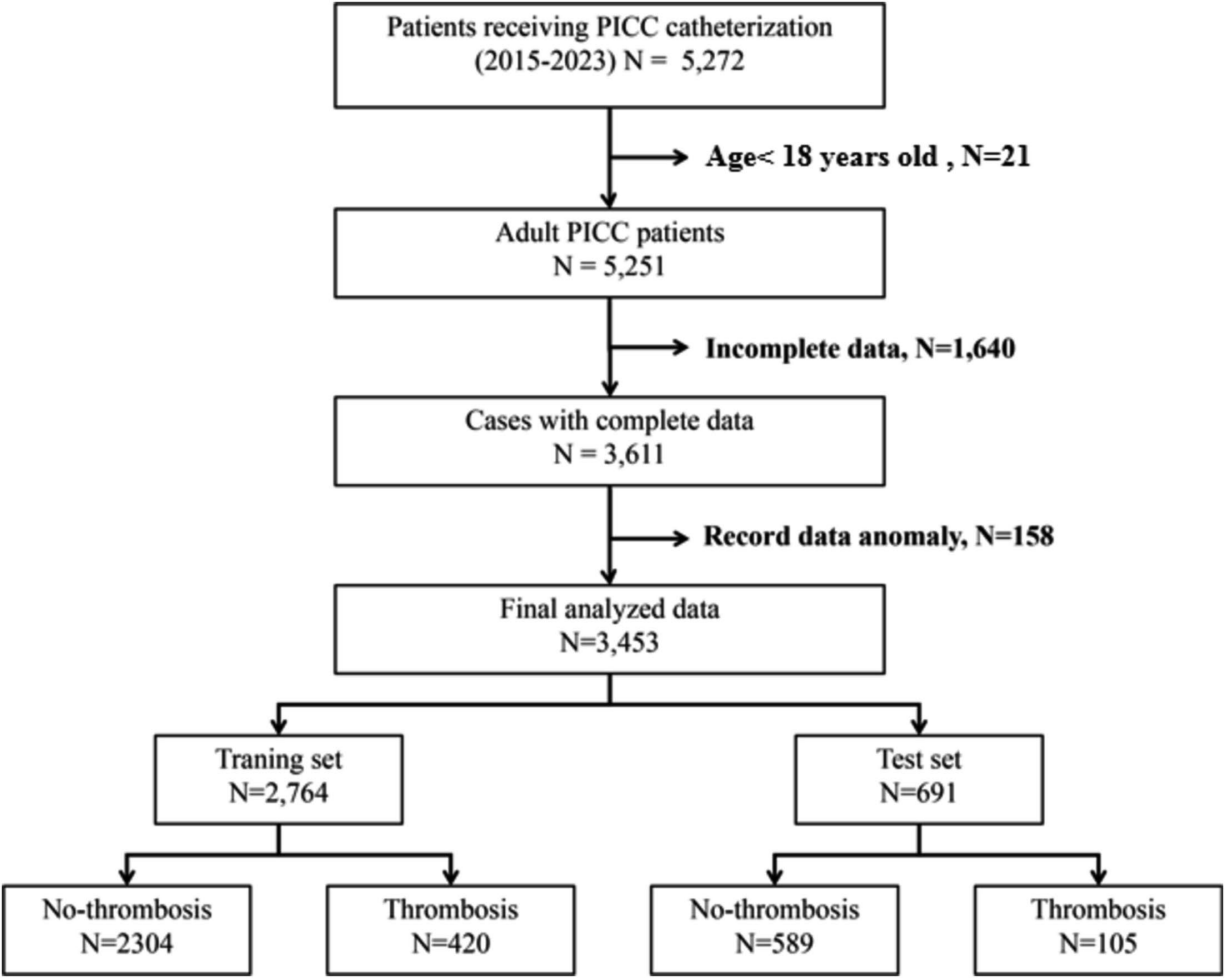
Data inclusion and exclusion flowchart.

From October 2015 to July 2023, clinical data for 5,272 patients who received PICC insertion treatment were gathered across 27 hospitals. In the data preparation phase, 1,815 patients were excluded due to issues with their records among other reasons, as depicted in Figure 1. Consequently, 3,453 patients who met the eligibility criteria were incorporated into the study. The demographic and clinical pathological characteristics of these patients are detailed in Table 1.

The age range of the patients included in the study was from 18 to 80 years, with a smaller proportion of males (32.7%) compared to females (67.3%). Among the patients with PICC, thrombosis occurred in 525 cases (15.2%). The majority, or 71.8%, of the patients undergoing PICC catheterization were diagnosed with stage III cancer or higher. Approximately 80% of these patients required long-term chemotherapy, utilizing treatments such as platinum-based drugs (37.7%), plant alkaloids (45.06%), and alkylating agents (34.1%). A total of 401 patients necessitated catheter removal due to recurrent thrombotic complications, whereas only 124 patients were able to continue using the catheter after receiving nursing care.

### 3.2 Variable selection

Through statistical analysis, we found that factors such as gender, age, height, education, primary diagnosis upon admission, activated partial prothrombin time, albumin, method of catheter fixation, type of pre-filled sealing fluid, type of connector, catheter opening, type of dressing, and certain chemotherapy drugs (such as platinum, anthracyclines, alkylating agents, and plant alkaloids) exhibited highly significant statistical differences (*p* < 0.001). Additionally, variables like weight, platelet count, prothrombin time, fibrinogen, history of deep vein catheterization, hypertension, puncture site, puncture complications, type of sealing fluid, and method of catheter tip positioning showed statistically significant correlations (*p* < 0.05), as detailed in Table 2.

**Table 2:** Univariate analysis of the risk factors of PICC-RVT.

Variable	Univariate analysis		
	Odds ratio	95% CI	*p*-value
Demographic characteristics			
Gender	0.3921	−1.1273–0.7452	<0.001
Age (years)	0.0256	0.0172–0.0341	<0.001
Height (cm)	0.0379	0.0265–0.0493	<0.001
Weight (kg)	0.0106	0.0027–0.0185	0.009
Education	0.8157	−0.2854–0.1222	<0.001
Tumor staging	0.9982	−0.0881–0.0845	0.967
Chemotherapy cycle	1.0083	−0.0217–0.0382	0.588
Main diagnosis of admission	0.9273	−0.1055–0.0455	<0.001
Laboratory indicators			
White blood cell (10×10^9^/L)	0.0224	0.0528–0.0080	0.149
Hemoglobin (g/L)	0.0014	−0.0011–0.0040	0.290
Platele (10^9^/L)	0.0015	−0.0026–0.0003	0.008
Prothrombin time (s)	−0.0338	−0.0660–0.0015	0.040
D-dimer (mg/L)	0.0078	−0.0112–0.0269	0.419
Fibrinogen (g/L)	0.0851	0.0181–0.1522	0.013
Activated partial prothrombin time (s)	−0.0391	−0.0547–0.0234	<0.001
Albumin (g/L)	0.0284	0.0207–0.0361	<0.001
C reactive protein (mg/L)	0.0011	−0.0022–0.0046	0.498
Past medical history			
Hypertension	0.7599	−0.5366–0.0125	0.040
Diabetes	0.8892	−0.4751–0.2403	0.519
Coronary heart disease	0.3469	−2.4933–0.3757	0.148
Past deep vein thrombosis and thrombophlebitis	1.8902	−0.0915–1.3648	0.086
History of deep vein catheterization	1.4518	0.0293–0.7163	0.033
Other	0.9810	−0.0815–0.0432	0.547
Puncture related information			
Puncture site (left vs. Right)	0.8344	−0.3471–0.0149	0.032
Number of punctures	1.3533	−0.0539–0.6591	0.096
Position of catheter tip	1.0062	−0.0466–0.05898	0.819
Tube placement method	0.6558	−0.6749–0.1691	0.001
Puncture blood vessel	0.9076	−0.3258–0.1319	0.406
Puncture complications	0.4864	−1.2608–0.1807	0.008
StatLock fixed PICC	0.6315	0.2405–0.8409	<0.001
Type of disinfectant	0.9753	−0.2517–0.2017	0.828
Pre-filled tube sealing fluid	0.4418	−1.0785–0.5553	<0.001
Dressing type	0.1908	−1.7507–1.5626	<0.001
Conduit related information			
Catheter vessel diameter ratio	1.0466	−0.1091–2.2023	0.076
Type of sealing fluid	2.0601	0.1550–1.2906	0.012
Reason for catheter retention	0.8904	−0.2937–0.0616	0.200
Connector type	1.7076	0.3529–0.7173	<0.001
Positioning method of catheter tip	1.3783	0.1104–0.5314	0.002
PICC catheter opening	0.5245	−0.7730–0.5175	<0.001
PICC model	1.1949	−0.1117–0.4678	0.228
Number of PICC cavities	0.0010	−89.2205–75.3853	0.869
Chemotherapy drugs			
Platinum drugs	1.9734	0.4901–0.8694	<0.001
Anthracycline drugs	0.4390	−1.0655–0.5812	<0.001
Alkylating agents	0.4382	−1.0587–0.5913	<0.001
immune modulators	0.0001	−290.7380–272.7910	0.950
Targeted drugs	0.7751	−0.5170–0.0075	0.056
Plant alkaloids	0.6636	−0.6052–0.2151	<0.001
Anti-metabolite drugs	1.2743	0.0208–0.4640	0.032
Corticosteroid medications	0.7678	−1.2081–0.6796	0.583
Topoisomerase Drugs	1.4919	0.1240–0.6761	0.004
Other chemotherapy drugs	0.8329	−0.4583–0.0928	0.193

Following the statistical correlation analysis, two models were developed for each outcome, differentiated by the number of predictive features. The initial models incorporate variables demonstrating exceptionally high statistical significance (p < 0.001), while the subsequent models include variables with significant statistical relevance (*p* < 0.05). Any variable with a *p*-value exceeding 0.05 was excluded, concentrating the analysis on predictors with substantial statistical backing. This meticulous approach to selecting input variables enhances the models’ predictive accuracy.

### 3.3 Model development and comparison

**Figure 2:**
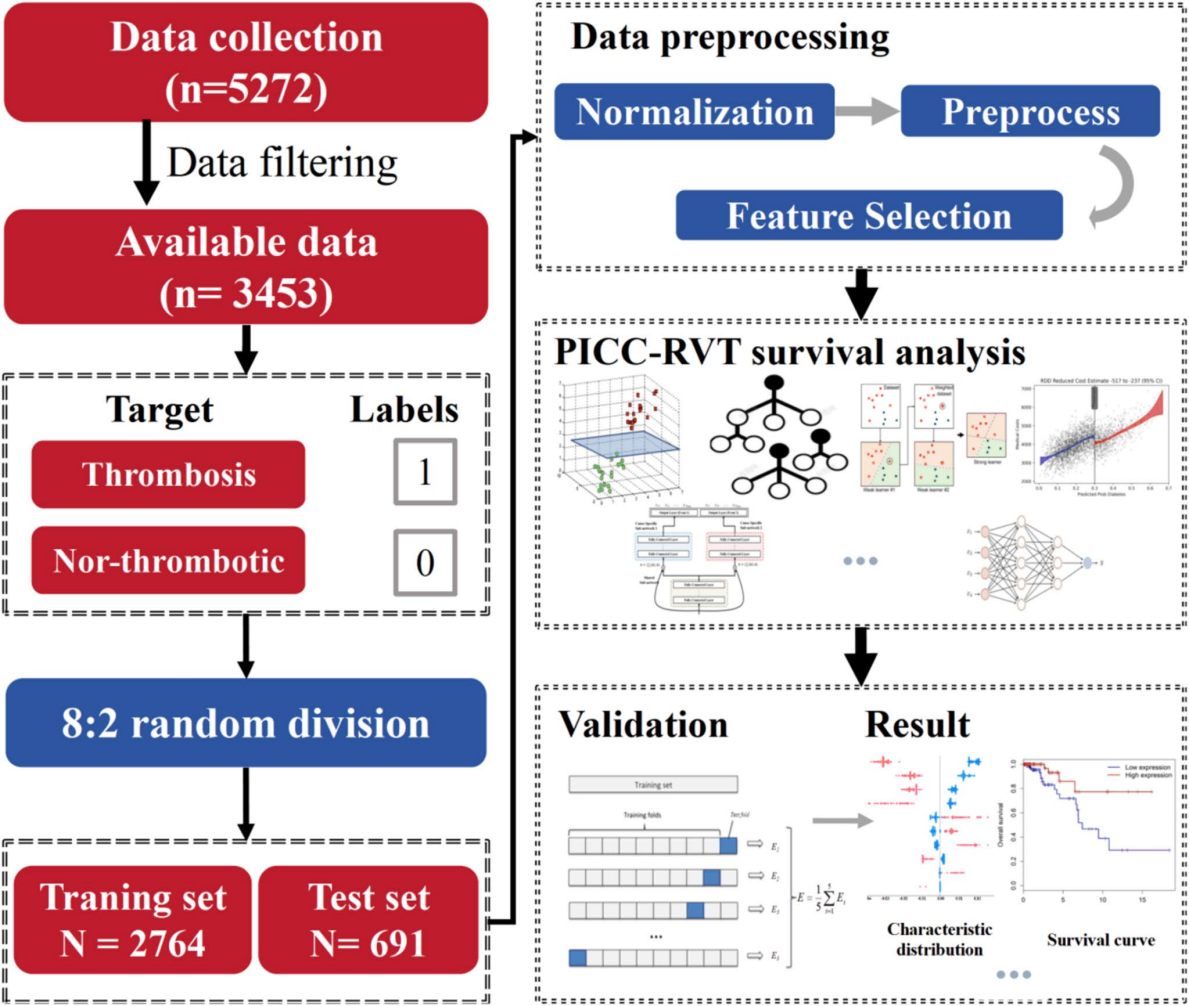
PICC-RVT survival analysis model establishment process.

To evaluate the performance and stability range of all predictive models for PICC-RVT occurrence, we divided the dataset into a training set and a test set in an 8:2 ratio. Additionally, we applied data preprocessing, feature selection, and machine learning algorithms. We employed three normalization methods (MinMax, Z-Score, Mean), two feature reduction techniques (PCA, PCC), and four feature selection methods (RFE, Relief, ANOVA, KW). Finally, we determined the highest concordance index and comprehensive B score for each model through five-fold cross-validation (algorithm workflow is illustrated in Figure 2). The implementation example code for the algorithms is detailed in Supplementary Table S2. The choice of data processing method and final hyperparameters for each algorithm were manually adjusted to achieve the highest concordance index and comprehensive B score, tailored to the specific dataset and feature variability of the study.

#### 3.3.1 Cause-specific deep learning prediction

In our study, we undertook extensive hyperparameter tuning using five-fold cross-validation, examining a range of hyperparameters including hidden layers (1 to 5 layers), nodes per layer (16, 32, 64, 128, 256), and dropout rates (0.1, 0.2, 0.3, 0.4, 0.5). After numerous model attempts, we ultimately selected the optimal training hyperparameters as shown in Table 3. For example, in our dataset, the DeepSurv model exhibited peak performance with 3 hidden layers, 64 nodes per layer, and a dropout rate of 0.2. These hyperparameters were chosen for their ability to maximize the concordance index and minimize the integrated Brier score, thereby ensuring the best possible model performance.

**Table 3:** Deep learning-based survival models and their performance and stability measures.

Models (Parameter)	Hyperparameters	C-Index Mean SD)	B-score Mean SD)	ICC (95%CI)
DeepSurv (26/16)	Layers = 3 Nodes per layer = 64 Dropout = 0.2 Batch size = 64 Learning rate = 0.01	**0.95 (0.007)**^a^/0.759 (0.012)	0.032 (0.081)/0.042 (0.091)	0.983 (0.931–1.00)/0.973 (0.921–0.99)
DeepHit (26/16)	Layers = 3 Nodes per layer = 64 Dropout = 0.2 Batch size = 64 Learning rate = 0.01	0.948 (0.012)/0.723 (0.012)	0.061 (0.052)/0.071 (0.062)	0.974 (0.908–1.00)/0.964 (0.898–0.99)
Cox-Time (26/16)	Layers = 3 Nodes per layer = 64 Dropout = 0.2 Batch size = 64 Learning rate = 0.001	0.949 (0.008)/0.741 (0.014)	0.045 (0.044)/0.055 (0.054)	0.984 (0.914–1.00)/0.974 (0.904–0.99)
MP-RSF (26/16)	N (trees) = 1,000 Features considered for best split = √N	0.772 (0.012)/0.741 (0.016)	0.064 (0.023)^b^/0.064 (0.033)	0.942 (0.833–1.00)/0.932 (0.823–0.99)
MP-AdaBoost (26/16)	weak learners = 1,000 Learning rate = 0.001 Features considered for best split = √N	0.731 (0.017)/0.723 (0.077)	0.067 (0.025)/0.077 (0.035)	0.936 (0.712–1.00)/0.926 (0.702–0.99)
ThresReg (26/16)	Regularization strength = 0.1 Learning rate = 0.001	0.707 (0.023)/0719 (0.063)	0.076 (0.027)/0.075 (0.037)	0.931 (0.751–1.00)/0.921 (0.741–0.99)
MP-LogitR (26/16)	Regularization type: Lasso/L2 Regularization strength = 0.1 Learning rate = 0.001 Convergence threshold = 1e-6	0.719 (0.022)/0.722 (0.042)	0.075 (0.031)/0.074 (0.041)	0.927 (0.732–1.00)/0.917 (0.722–0.99)

C-index, Concordance index; B-score, Brier score; ICC, Intraclass correlation coefficient; CI, confidence interval. Texts in bold are indicative of the highest performance measure or stability evaluator, i.e., concordance index, integrated B-score, and ICC.

a Not statistically significant at probability values below 5% (one-way analysis of variance).

b Statistically significant at probability values below 5% (one-way analysis of variance).

**Figure 3:**
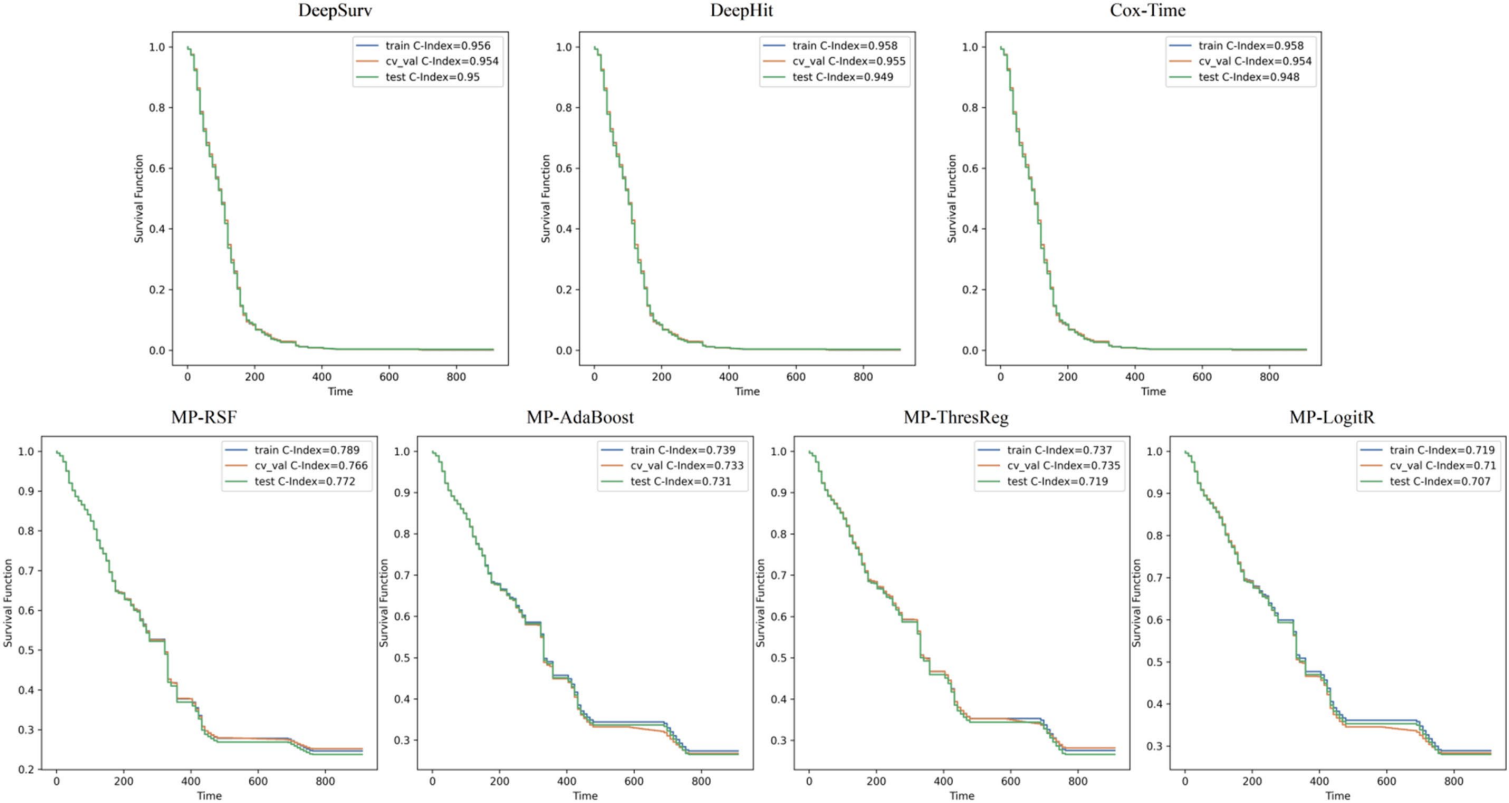
Seven survival analysis models based on 26-parameter survival curves.

The training results for the 26-feature model are illustrated in Figure 3, while the 16-feature model results are shown in Supplementary Figure S1. The results indicate that the three deep learning models (DeepSurv, DeepHit, Cox-Time) have strong predictive capabilities, with the DeepSurv model exhibiting particularly outstanding discriminative performance. The DeepSurv model achieved a high concordance index (C-index) of 0.95 in the 26-feature model, followed by the Cox-Time and DeepHit models. Overall, the predictive performance of the 26-feature model was superior to that of the 16-feature model (as shown in Appendix Figure 1). Regarding the Brier score, the DeepSurv model achieved a score of 0.032, with an average standard deviation of 0.081, which is slightly higher than the other deep learning algorithms but both scores are well below the critical value of 0.25.

#### 3.3.2 Cause-specific machine learning prediction

In machine learning, we compared four machine learning methods (RSF, AdaBoost, ThresReg, LogitR) with both 26 parameters and 16 parameters. After manual tuning, each model exhibited improved discriminative power. Among them, the RSF model performed the best, with C-Index scores of 0.772 and 0.741, respectively. The integrated Brier score for both the 26-parameter and 16-parameter models was 0.064, reflecting similar accuracy of the predicted survival function to the actual survival status for both models.

### 3.4 Important variables of prediction models

**Figure 4:**
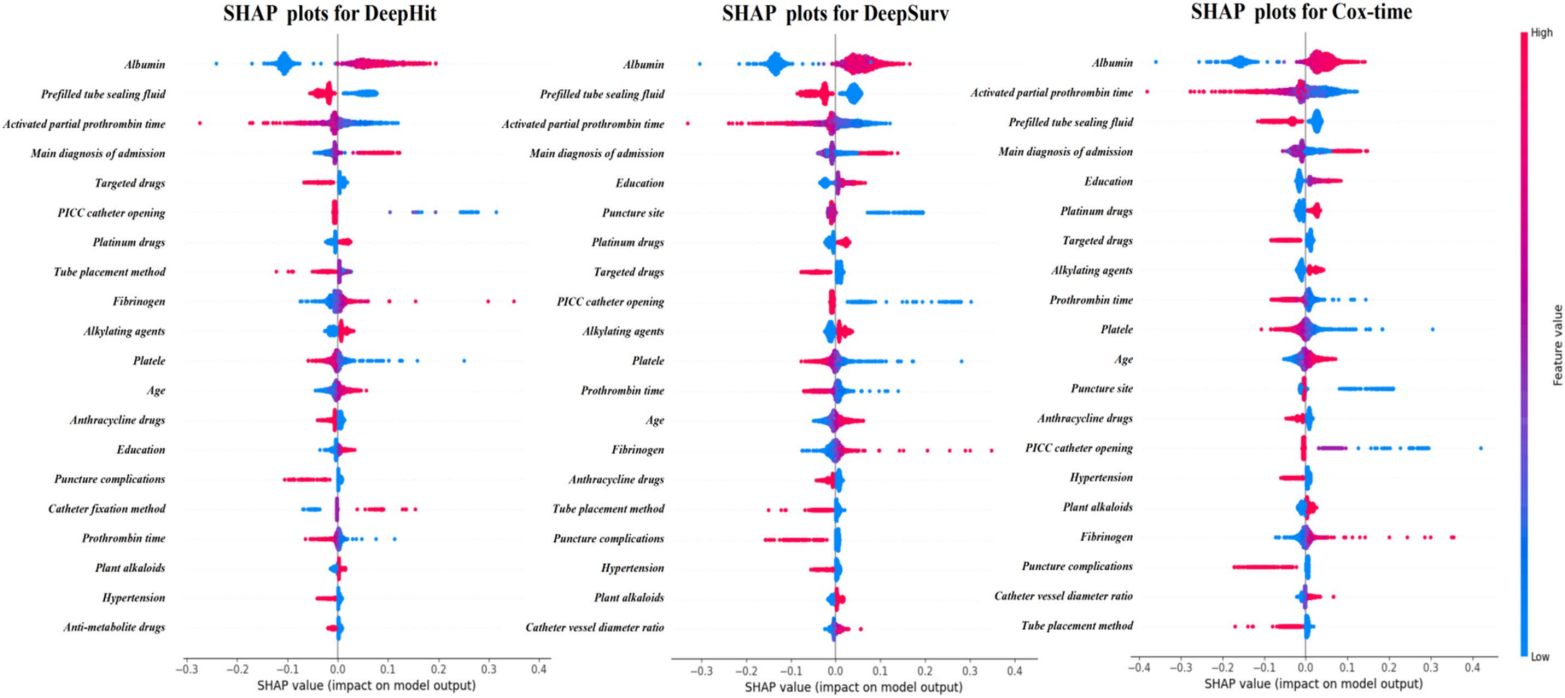
SHAP diagram of deep learning algorithm.

Figure 4 displays SHAP (Shapley additive Explanations) graphs for three deep learning methods, detailing the comparative importance of each feature (33, 34). The results reveal that albumin, type of pre-filled locking solution, and activated partial thromboplastin time are the strongest predictors in the deep learning predictive models.

The top 8 important variables for each predictive model show that regardless of the model used, albumin and the type of sealing solution are the most critical variables in predicting PICC-RVT (Tables 4, 5), particularly the type of sealing solution involving heparin saline. Regardless of the model used, a patient’s primary diagnosis, the method of PICC placement, targeted therapies, and platinum-based drugs consistently exhibit high variable importance.

**Table 4:** Top 8 important variables of deep learning prediction models.^*^

Rank	Models		
	DeepSurv	DeepHit	Cox-Time
1	Albumin	Albumin	Albumin
2	Pre-filled tube sealing fluid	Activated partial prothrombin time	Pre-filled tube sealing fluid
3	Activated partial prothrombin time	Pre-filled tube sealing fluid	Activated partial prothrombin time
4	Main diagnosis of admission	Targeted drugs	Main diagnosis of admission
5	Targeted drugs	Platinum drugs	Targeted drugs
6	PICC catheter opening	PICC catheter opening	PICC catheter opening
7	Platinum drugs	Tube placement method	Platinum drugs
8	Tube placement method	Main diagnosis of admission	Tube placement method

* Variable importance is determined by Shap value.

**Table 5:** Top 8 important variables of machine learning prediction models.^*^

Rank	Models			
	MP-RSF	MP-AdaBoos	ThresReg	MP-LogitR
1	Albumin (rise)	Albumin (rise)	Albumin (rise)	Albumin (rise)
2	Pre-filled tube sealing fluid (yes)	Platele	Main diagnosis of admission	Main diagnosis of admission
3	Activated partial prothrombin time	Targeted drugs	Normal saline	Normal saline
4	Blood system	Main diagnosis of admission	Platele	Targeted drugs
5	Targeted drugs	Alkylating agents	Age	Activated partial prothrombin time
6	Platinum drugs	Front opening	Targeted drugs	Education
7	Front opening	Age	Education	Alkylating agents
8	Age	Prothrombin time	Front opening	Age

* Variable importance is determined by the frequency of selection of the variable as a decision node in the logistic model.

## 4 Discussion

Peripherally Inserted Central Catheters (PICCs) are an essential part of contemporary medical practice, especially for patients requiring prolonged intravenous therapy (35). These catheters offer multiple benefits: they minimize the risk of infections, allow for extended usage, enhance patient comfort, and reduce the frequency of venipunctures. Such advantages establish PICCs as the preferred method for administering long-term antibiotics, chemotherapy, nutritional support, and managing intricate medication regimens. By alleviating patient discomfort and reducing the risk of complications, PICCs significantly contribute to improved treatment outcomes, quicker patient recovery, and decreased healthcare costs (36, 37).

Despite their considerable benefits, the use of PICCs is associated with certain risks, including complications like PICC-related venous thrombosis (PICC-RVT). PICC-RVT may manifest as pain, swelling, and catheter malfunction; in severe cases, it can escalate to life-threatening thromboembolic events such as pulmonary embolism. Accurate prediction of PICC-RVT is therefore crucial. Effective risk prediction enables healthcare professionals to take preventive measures, such as choosing the optimal catheter type and site, regularly monitoring the patient’s blood status, and, if necessary, administering anticoagulant or thrombolytic therapy (4).

In modern medical disease management, accurate and timely prognostic predictions are vital for enhancing patient outcomes and quality of life. While traditional inferential statistical models rely on historical data and statistical inference, the advent of big data and increased computational capabilities has favored machine learning models for prognostication. Machine learning excels in handling complex datasets and identifying latent patterns and correlations, offering dynamic prognostic probabilities that adjust over time (16). This evolving approach is crucial, as the effectiveness of predictive models can wane over extended periods (28). Incorporating event timing into these models not only provides more accurate risk assessments but also captures the changing nature of risk over time, making it particularly suited for the long-term monitoring of complications associated with PICC use.

Recent studies have demonstrated the potential of machine learning models in predicting PICC-related venous thrombosis (PICC-RVT) risks, integrating various predictive factors and time-to-event data to enhance predictive accuracy. Our research builds on these findings by incorporating seven different machine learning algorithms, including both traditional and deep learning models, to evaluate their effectiveness in assessing PICC-RVT risks. Supplementary Table S4 provides a summary of related works from the past 5 years that are pertinent to this study.

The evaluation revealed that deep learning models, on average, outperformed machine learning models in terms of predictive performance, with a higher average c-index (0.949 vs. 0.732) and a lower average comprehensive Brier score (0.046 vs. 0.093). Among these, the DeepSurv model stood out, showcasing superior performance and exceptional accuracy in risk assessment, evidenced by its c-index of 0.95 and an excellent Brier score of 0.032, indicating minimal prediction error. Moreover, the Cox-Time model displayed the highest ICC value at 0.984, suggesting slightly greater reliability compared to the DeepSurv model. However, the DeepSurv model’s accuracy and relative reliability in predicting PICC-RVT, coupled with its outstanding prediction stability, could enhance its popularity in certain clinical scenarios. Additionally, it was observed that the model’s stability varies with the number of predictors; specifically, Cox-Time exhibits the highest intra-class correlation coefficient with 16 predictors (ICC: 0.974), whereas DeepSurv achieves the greatest stability with 26 predictors (ICC: 0.983).

In this study, we found that models equipped with 26 parameters generally surpassed those with only 16 parameters in predictive performance. This improvement can be attributed to the fact that a greater number of parameters enhances the model’s learning capacity, allowing it to grasp more complex data relationships, which is crucial for handling multi-dimensional data. However, the increase in parameters also raises the risk of overfitting, characterized by high performance on training data but poor generalization to new data (38). This is especially true for models like ThresReg and MP-LogitR, where analyzing the relevance of each parameter is vital to prevent them from negatively impacting prediction accuracy. In the context of deep learning, techniques such as Dropout have been shown to mitigate the effects of irrelevant parameters, effectively reducing overfitting risks while still benefiting from a higher parameter count (39). Thus, applying appropriate measures during the training of deep learning models can control overfitting, significantly improving their capability to predict the risk of PICC-RVT.

Through an in-depth analysis of deep learning and machine learning models in this study, it was discovered that certain variables, notably albumin levels and the type of locking solution used, play a pivotal role in predicting the risk of venous thrombosis associated with PICC. Albumin levels, in particular, are of paramount importance. As the most abundant protein in plasma, albumin not only reflects a patient’s nutritional status and inflammatory response but also is vital for maintaining intravascular colloid osmotic pressure (40). Low albumin levels can signify inflammation, malnutrition, or protein loss, factors that elevate thrombosis risk. In the context of PICC catheterization, hypoalbuminemia can increase blood viscosity, thereby heightening the risk of thrombus formation. Furthermore, the choice of locking solution, especially the use of heparin saline, is critical in mitigating the risk of PICC-RVT. Unlike saline, which is commonly used, heparin saline possesses anticoagulant properties that prevent thrombosis. Its action mechanism involves activating antithrombin, thus inhibiting blood clot formation and minimizing the interaction between PICC lines and endothelial cells, effectively reducing thrombosis risk (41). Recent studies and clinical practices have demonstrated that saline is as effective as heparinized saline in preserving catheter patency, avoiding complications associated with heparin use (42). Consequently, the effect of heparin saline on PICC-RVT risk warrants additional investigation.

The primary diagnosis of patients has been identified as a crucial factor influencing the risk of PICC-RVT. Pathological conditions such as malignancies, infections, and other chronic diseases can elevate thrombosis risk due to their inherent pathophysiological traits. In particular, certain cancers are known to increase blood coagulation, thereby heightening the risk of PICC-RVT (43). Additionally, the use of targeted therapies and platinum-based drugs significantly affects this risk, mainly due to their pharmacological effects and potential harm to endothelial cells. While targeted therapies aim to attack tumor cells, they may also impair endothelial functions, fostering a procoagulant environment and enhancing thrombus formation likelihood (44). Similarly, platinum-based chemotherapy agents can damage blood vessels and encourage coagulation, increasing the probability of PICC-RVT (45, 46). Consequently, these treatments necessitate closer monitoring of PICC lines and vigilance for signs of thrombosis during therapy.

This investigation employed retrospective multi-center cohort designs, utilizing both deep learning and traditional machine learning models to predict the risk associated with PICC-RVT. Although the study benefited from extensive patient data, the exclusion of 1,815 patients due to incomplete records may have impacted the findings. While seven survival analysis models were evaluated, further improvements in variable selection and model optimization are needed to enhance predictive performance and clinical relevance. To integrate these predictive models into real-time clinical workflows, we propose embedding the models into electronic health record (EHR) systems for automatic data extraction and real-time risk prediction, developing real-time alert systems to notify healthcare providers of high-risk patients, implementing continuous model updates with new patient data, creating user-friendly interfaces and visualization tools for clear presentation of risk predictions, facilitating interdisciplinary collaboration between data scientists, clinicians, and IT specialists, and providing regular training and support for clinical staff to ensure effective use and acceptance of the predictive tools.

A key limitation of this research is the lack of prospective clinical validation, which is crucial for confirming the practical effectiveness of the predictive models. Nonetheless, the study has demonstrated notable successes in forecasting PICC-RVT risk. The integration of deep learning and machine learning models has markedly refined the accuracy of survival probabilities and relative risk predictions for specific conditions. These models have also facilitated the identification of critical factors influencing PICC-RVT risk, such as albumin levels and the type of locking solution used.

Additionally, the study highlighted the influence of targeted therapies and platinum-based drugs on thrombosis risk, underscoring their importance in clinical assessments. Through meticulous feature selection and model refinement, the predictive models’ accuracy and reliability were significantly improved, providing a valuable and practical tool for clinical risk assessment. This breakthrough enhances the capability for early detection and intervention for high-risk individuals, greatly supporting clinical decisions concerning the management of PICC lines. Such advancements not only promise to optimize individual patient care but also have the potential to inform broader public health strategies aimed at preventing serious complications associated with PICC use.

## 5 Conclusion

This research evaluated seven time-to-event algorithms designed to predict the risk of PICC-RVT. Each model demonstrated high levels of prediction accuracy, calibration, and stability. Deep learning technologies, in particular, surpassed traditional machine learning methods in forecasting medical outcomes. Among them, the DeepSurv model was notably effective in distinguishing between high-risk and low-risk patients, thus facilitating more informed treatment decisions. Key predictors of PICC-RVT risk identified include albumin levels, the type of pre-filled sealant, and partial thromboplastin time. Moving forward, it will be crucial to further validate these models to enhance their predictive accuracy and clinical utility. Continued efforts to refine model performance and identify crucial predictors are essential for improving the clinical application viability of these models, ultimately enhancing patient outcomes in the management of PICC.

## Data Availability

The raw data supporting the conclusions of this article will be made available by the authors, without undue reservation.

## References

[1] Aryal M Pathak R Giri S Bhatt VR. Recent trends in incidence and financial impact of peripherally inserted central catheter (PICC)-associated upper extremity deep vein thrombosis (DVT): Data from National Inpatient Sample. Blood. (2017) 130:3359. doi: 10.1182/blood.V130.Suppl_1.3359.3359

[2] Trerotola SO Stavropoulos SW Mondschein JI Patel AA Fishman N Fuchs B. Triple-lumen peripherally inserted central catheter in patients in the critical care unit: prospective evaluation. Radiology. (2010) 256:312–20. doi: 10.1148/radiol.10091860, PMID: 20574104

[3] Pan L Zhao Q Yang X. Risk factors for venous thrombosis associated with peripherally inseRVTed central venous catheters. Int J Clin Exp Med. (2014) 7:5814–9. PMID: 25664112 PMC4307559

[4] Flinterman LE van FJM Rosendaal FR Doggen CJM. Current perspective of venous thrombosis in the upper extremity. J Thromb Haemost. (2008) 6:1262–6. doi: 10.1111/j.1538-7836.2008.03017.x, PMID: 18485082

[5] Kang J Chen W Sun W Ge R Li H Ma E. Peripherally inserted central catheter-related complications in cancer patients:a prospective study of over 50,000 catheter days. J Vasc Access. (2017) 18:153–7. doi: 10.5301/jva.5000670, PMID: 28218366

[6] Kreuziger LB Jaffray J Carrier M. Epidemiology, diagnosis, prevention and treatment of catheter-related thrombosis in children and adults. Thromb Res. (2017) 157:64–71. doi: 10.1016/j.thromres.2017.07.002, PMID: 28710972

[7] Bzdok D Altman N Krzywinski M. Statistics versus machine learning. Nat Methods. (2018) 15:233–4. doi: 10.1038/nmeth.4642, PMID: 30100822 PMC6082636

[8] Song X Lu H Chen F Bao Z Li S Li S. A longitudinal observational retrospective study on risk factors and predictive model of PICC associated thrombosis in cancer patients. Sci Rep. (2020) 10:10090–13. doi: 10.1038/s41598-020-67038-x, PMID: 32572092 PMC7308336

[9] Wang KL Yap ES Goto S Zhang S Siu CW Chiang CE. The diagnosis and treatment of venous thromboembolism in Asian patients. Thromb J. (2018) 16:1–12. doi: 10.1186/s12959-017-0155-z, PMID: 29375274 PMC5774147

[10] Lin S Zhu N Zhang Y Du L Zhang S. Development and validation of a prediction model of catheter-related thrombosis in patients with cancer undergoing chemotherapy based on ultrasonography results and clinical information. J Thromb Thrombolysis. (2022) 54:480–91. doi: 10.1007/s11239-022-02693-7, PMID: 35972592 PMC9553810

[11] Liu S Zhang F Xie L Wang Y Xiang Q Yue Z. Machine learning approaches for risk assessment of peripherally inserted central catheter-related vein thrombosis in hospitalized patients with cancer. Int J Med Inform. (2019) 129:175–83. doi: 10.1016/j.ijmedinf.2019.06.001, PMID: 31445252

[12] Lin Y Zeng Z Lin R Zheng J Liu S Gao X. The Caprini thrombosis risk model predicts the risk of peripherally inserted central catheter-related upper extremity venous thrombosis in patients with cancer. J Vasc Surg Venous Lymp Dis. (2021) 9:1151–8. doi: 10.1016/j.jvsv.2020.12.075, PMID: 33383236

[13] Yue J Zhang Y Xu F Mi A Zhou Q Chen B. A clinical study of peripherally inserted central catheter-related venous thromboembolism in patients with hematological malignancies. Sci Rep. (2022) 12:9871. doi: 10.1038/s41598-022-13916-5, PMID: 35701467 PMC9197841

[14] Chopra V Kaatz S Conlon A Paje D Grant PJ Rogers MAM. The Michigan risk score to predict peripherally inserted central catheter-associated thrombosis. J Thromb Haemost. (2017) 15:1951–62. doi: 10.1111/jth.13794, PMID: 28796444

[15] Haider H Hoehn B Davis S Greiner R. Effective ways to build and evaluate individual survival distributions. J Mach Learn Res. (2020) 21:3289–351.

[16] Adeoye J Hui L Koohi-Moghadam M Tan JY Choi SW Thomson P. Comparison of time-to-event machine learning models in predicting oral cavity cancer prognosis. Int J Med Inform. (2022) 157:104635. doi: 10.1016/j.ijmedinf.2021.104635, PMID: 34800847

[17] Hao L Kim J Kwon S Ha ID. Deep learning-based survival analysis for high-dimensional survival data. Mathematics. (2021) 9:1244. doi: 10.3390/math9111244

[18] Ryu JY Lee MY Lee JH Lee BH Oh KS. DeepHIT: a deep learning framework for prediction of hERG-induced cardiotoxicity. Bioinformatics. (2020) 36:3049–55. doi: 10.1093/bioinformatics/btaa075, PMID: 32022860

[19] Rahman SA Walker RC Maynard N Nigel Trudgill Crosby T Cromwell DA. The AUGIS survival predictor: prediction of long-term and conditional survival after esophagectomy using random survival forests. Ann Surg. (2022) 277:267–74. doi: 10.1097/SLA.0000000000004794, PMID: 33630434 PMC9831040

[20] Nusinovici S Tham YC Yan MYC Ting DSW Li J Sabanayagam C. Logistic regression was as good as machine learning for predicting major chronic diseases. J Clin Epidemiol. (2020) 122:56–69. doi: 10.1016/j.jclinepi.2020.03.002, PMID: 32169597

[21] Serviá L Montserrat N Badia M Llompart-Pou JA Barea-Mendoza JA Chico-Fernández M. Machine learning techniques for mortality prediction in critical traumatic patients: anatomic and physiologic variables from the RETRAUCI study. BMC Med Res Methodol. (2020) 20:1–12. doi: 10.1186/s12874-020-01151-3, PMID: 33081694 PMC7576744

[22] Wang Q Wang L. The nonlinear effects of population aging, industrial structure, and urbanization on carbon emissions: a panel threshold regression analysis of 137 countries. J Clean Prod. (2021) 287:125381. doi: 10.1016/j.jclepro.2020.125381

[23] Nagpal C Li X Dubrawski A. Deep survival machines: fully parametric survival regression and representation learning for censored data with competing risks. IEEE J Biomed Health Inform. (2021) 25:3163–75. doi: 10.1109/JBHI.2021.3052441, PMID: 33460387

[24] Katzman JL Shaham U Cloninger A Bates J Jiang T Kluger Y. DeepSurv: personalized treatment recommender system using a cox proportional hazards deep neural network. BMC Med Res Methodol. (2018) 18:24. doi: 10.1186/s12874-018-0482-1, PMID: 29482517 PMC5828433

[25] Ishwaran H Kogalur UB. Consistency of random survival forests. Stat Prob Lett. (2010) 80:1056–64. doi: 10.1016/j.spl.2010.02.020, PMID: 20582150 PMC2889677

[26] Rozgic V Xiao B Katsamanis A Baucom B. Georgiou P. G. Narayanan S. (2011). “Estimation of ordinal approach-avoidance labels in dyadic interactions: Ordinal logistic regression approach.” in *IEEE International Conference on Acoustics. IEEE*.

[27] Lee C. Machine Learning Frameworks for Data-Driven Personalized Clinical Decision Support and the Clinical Impact[M]. University of California, Los Angeles (2021).

[28] Kvamme H Borgan Ø Scheel I. Time-to-event prediction with neural networks and cox regression (2019). doi: 10.48550/arXiv.1907.00825

[29] Nizam R Karim ZA Rahman AA Sarmidi T. Financial inclusiveness and economic growth: new evidence using a threshold regression analysis. Econ Res. (2020) 33:1465–84. doi: 10.1080/1331677X.2020.1748508

[30] Maryam S Kimber AC Javid S. Multivariable prognostic model for dialysis patients with end stage renal disease. Saudi Med J. (2021) 42:714–20. doi: 10.15537/smj.2021.42.7.20210082, PMID: 34187914 PMC9195532

[31] Sharkansky S. Discrete-Time Threshold Regression for Survival Data with Time-Dependent Covariates (Doctoral dissertation) (2015).

[32] Turney P. Technical note: Bias and the quantification of stability. Mach Learn. (1995) 20:23–33. doi: 10.1023/A:1022682001417

[33] Antwarg L Miller RM Shapira B Rokach L. Explaining anomalies detected by autoencoders using Shapley additive explanations. Expert Syst Appl. (2021) 186:115736. doi: 10.1016/j.eswa.2021.115736

[34] Slack D. Hilgard S. Jia E. Singh S. Lakkaraju H. (2020). “Fooling lime and shap: adversarial attacks on post hoc explanation methods.” in *Proceedings of the AAAI/ACM Conference on AI, Ethics, and Society*. pp. 180–186.

[35] Bosch FTM Nisio MD Büller HR van Es N. Diagnostic and therapeutic management of upper extremity deep vein thrombosis. J Clin Med. (2020) 9:2069. doi: 10.3390/jcm9072069, PMID: 32630244 PMC7408847

[36] Liem TK Yanit KE Moseley SE Landry GJ Deloughery TG Rumwell CA. Peripherally inserted central catheter usage patterns and associated symptomatic upper extremity venous thrombosis. J Vasc Surg. (2012) 55:761–7. doi: 10.1016/j.jvs.2011.10.005, PMID: 22370026

[37] Zochios V Umar I Simpson N Jones N. Peripherally inserted central catheter (PICC)-related thrombosis in critically ill patients. J Vasc Access. (2014 Sep-Oct) 15:329–37. doi: 10.5301/jva.5000239, PMID: 24811591

[38] Pavlitskaya S Oswald J Zöllner J M. (2022). “Measuring overfitting in convolutional neural networks using adversarial perturbations and label noise”. in *2022 IEEE symposium series on computational intelligence (SSCI)*. IEEE. pp. 1551–1559.

[39] Garbin C Zhu X Marques O. Dropout vs. batch normalization: an empirical study of their impact to deep learning. Multimed Tools Appl. (2020) 79:12777–815. doi: 10.1007/s11042-019-08453-9

[40] Yao W Zhang K Lv Q Deng Z Ding W. D-dimer-albumin ratio (DAR) as a new biomarker for predicting preoperative deep vein thrombosis after geriatric hip fracture patients. J Orthop Surg Res. (2023) 18:645. doi: 10.1186/s13018-023-04139-z, PMID: 37653556 PMC10470167

[41] Scamuffa MC Morano SG Serrao A Bruzzese A Stocchi F Santoro C. PICC-related upper deep venous thrombosis in patients with hematological malignancies. Management of anticoagulant therapy according to the platelet count. J Thromb Thrombolysis. (2020) 49:426–30. doi: 10.1007/s11239-020-02040-8, PMID: 31981040

[42] Zhong L Wang HL Xu B Yuan Y Wang X Zhang YY. Normal saline versus heparin for patency of central venous catheters in adult patients-a systematic review and meta-analysis. Crit Care. (2017) 21:1–9. doi: 10.1186/s13054-016-1585-x, PMID: 28063456 PMC5219914

[43] Falanga A Marchetti M Vignoli A. Coagulation and cancer: biological and clinical aspects. J Thromb Haemost Jth. (2013) 11:223–33. doi: 10.1111/jth.12075, PMID: 23279708

[44] Bozionellou V Mavroudis D Perraki M Papadopoulos S Apostolaki S Stathopoulos E. Trastuzumab administration can effectively target chemotherapy- resistant cytokeratin-19 messenger rna-positive tumor cells in the peripheral blood and bone marrow of patients with breast cancer. Clin Cancer Res. (2004) 10:8185–94. doi: 10.1158/1078-0432.CCR-03-0094, PMID: 15623593

[45] Hassan SA Palaskas N Kim P Iliescu C Lopez-Mattei J Mouhayar E. Chemotherapeutic agents and the risk of ischemia and arterial thrombosis. Curr Atheroscler Rep. (2018) 20:1–9. doi: 10.1007/s11883-018-0702-5, PMID: 29423705

[46] Wang D Lippard SJ. Cellular processing of platinum anticancer drugs. Nat Rev Drug Discov. (2005) 4:307–20. doi: 10.1038/nrd1691, PMID: 15789122

