# Supplementary material for: Integrating deep learning in public health: a novel approach to PICC-RVT risk assessment
Source: Front Public Health. 2025 Jan 7;12:1445425. doi: 10.3389/fpubh.2024.1445425 (PMC11747573; doi:10.3389/fpubh.2024.1445425)
Supplement: Supplementary file 1 [file Data_Sheet_1.docx]

**Table S1. Number of PICC patients in each hospital**

| **Hospital Name** | **Data contribution** | **Hospital Name** | **Data contribution** |
| --- | --- | --- | --- |
| Xiangya Hospital Central South University | 220 | Southern medical university nanfang hospital | 55 |
| Beijing Cancer Hospital | 197 | Qilu Hospital of Shandong University | 46 |
| The First Hospital of Shanxi Medical University | 35 | Affiliated Hospital of Guilin Medical College | 14 |
| Renmin Hospital of Wuhan University | 57 | Liuzhou people's Hospital | 42 |
| Guangxi Workers' Hospital | 22 | Qinzhou First People's Hospital | 263 |
| Guigang People's Hospital | 214 | Yulin First People's Hospital | 32 |
| Guilin People's Hospital | 19 | Beijing Hospital | 188 |
| Guangxi Zhuang Autonomous Region Ethnic Hospital | 25 | Beijing Chaoyang District Huanxing Knub Hospital | 2099 |
| Cancer Hospital Affiliated to Guangxi Medical University | 128 | The First Affiliated Hospital of Guangxi University of Traditional Chinese Medicine | 6 |
| Hunan Provincial Tumor Hospital | 769 | Zhanjiang Center People Hospital | 20 |
| Foshan first people hospital | 158 | Shiyan Taihe Hospital | 28 |
| Jieyang People's Hospital | 11 | Yulin Red Cross Hospital | 56 |
| Guangzhou No.1 People's Hospital | 78 | Zhongshan People's Hospital | 18 |
| Meizhou People's Hospital | 472 |  |  |

**Table S2. Algorithm code source**

| **Models** | **Code source** |
| --- | --- |
| DeepSurv | https://github.com/czifan/DeepSurv.pytorch |
| DeepHit | https://github.com/chl8856/DeepHit |
| Cox-Time | This code comes from the lifelines library in python |
| cs-MP-RSF | https://github.com/julianspaeth/random-survival-forest |
| cs-MP-AdaBoost | This code comes from the sklearn library in python |
| cs-ThresReg | This code comes from the sklearn library in python |
| cs-MP-LogitR | This code comes from the sklearn library in python |

**Table S3. Characteristics of a typical patient with thrombosis**

| **Feature** | **Value** | **Feature** | **Value** |
| --- | --- | --- | --- |
| **Gender** | Female | **Age (years)** | 56 |
| **Height (cm)** | 153 | **Educational Level** | High School |
| **Main diagnosis of admission** | Mammary glands | **Activated partial prothrombin time** | 29 |
| **Albumin (g/L)** | 40 | **Method of Catheter Fixation** | StatLock fixed PICC |
| **Type of Pre-filled Locking Solution** | Heparin Saline | **Type of Connector** | Positive Pressure Connector |
| **Catheter Opening** | Yes | **Type of Dressing** | Transparent Film |
| **Platinum-based Chemotherapy** | Yes | **Anthracyclines** | No |
| **Alkylating Agents** | Yes | **Plant Alkaloids** | No |
| **Weight (kg)** | 55 | **Platelet Count (10^9/L)** | 220 |
| **Prothrombin Time (s)** | 12.5 | **Fibrinogen (g/L)** | 3.2 |
| **History of Previous Deep Vein Catheterization** | No | **Hypertension** | Yes |
| **Puncture Site** | Right Arm | **Puncture Complications** | None |
| **Type of Sealing Solution** | Heparin | **Positioning method of catheter tip** | X-ray |

This table summarizes the characteristics of a typical patient with thrombosis complications. We transform these features into vectors and input them into predictive models to assess the risks and factors associated with thrombosis. During the prediction process, this patient has a unique characteristic: the number of days the catheter is retained. By modifying this feature value in the input vector, we can determine the patient's future probability of developing thrombosis.

**Table S4. Over the past five years, a summary of work related to this study.**

| **Year** | **Study** | **Methodology** | **Key Findings** |
| --- | --- | --- | --- |
| 2018 | Bzdok, D., Altman, N., & Krzywinski, M.^1^ | Statistical vs. Machine Learning Comparison | Compared traditional statistical methods with machine learning approaches for risk assessment, highlighting the superior accuracy of machine learning models in handling complex datasets. |
| 2018 | Wang, K. L., Yap, E. S., Goto, S., et al.^2^ | Clinical Guidelines Review | Addressed the diagnosis and treatment of venous thromboembolism in Asian patients, underscoring the regional differences in PICC-RVT incidence and management. |
| 2019 | Liu, S., Zhang, F., Xie, L., et al.^3^ | Machine Learning Models | Evaluated machine learning approaches for assessing PICC-related vein thrombosis risk, emphasizing the potential for enhanced predictive performance. |
| 2020 | Song, X., Lu, H., Chen, F., et al.^4^ | Retrospective Study with Machine Learning | Developed a predictive model for PICC-associated thrombosis in cancer patients using machine learning, demonstrating improved discriminative accuracy over traditional methods. |
| 2020 | Haider, H., Hoehn, B., Davis, S., et al.^5^ | Survival Analysis with Machine Learning | Discussed effective ways to build and evaluate individual survival distributions using machine learning, which is pertinent to our time-to-event data analysis. |
| 2021 | Lin, Y., Zeng, Z., Lin, R., et al.^6^ | Validation of Risk Models | Validated the Caprini thrombosis risk model for PICC-related upper extremity venous thrombosis in cancer patients, providing a basis for integrating machine learning into clinical practice. |
| 2021 | Hao, L., Kim, J. et al.^7^ | Deep Learning Survival Analysis | Presented a deep learning-based survival analysis for high-dimensional survival data, showcasing the flexibility and accuracy of deep learning models |
| 2022 | Yue, J., Zhang, Y.,et al.^8^ | Clinical Study with Risk Factor Analysis | Conducted a clinical study on PICC-RVT in patients with hematological malignancies, identifying key risk factors and improving prediction accuracy. |
| 2022 | Adeoye, J., Hui, L.,et al.^9^ | Comparison of Time-to-Event Models | Compared time-to-event machine learning models for cancer prognosis prediction, highlighting the advantages of deep learning algorithms. |


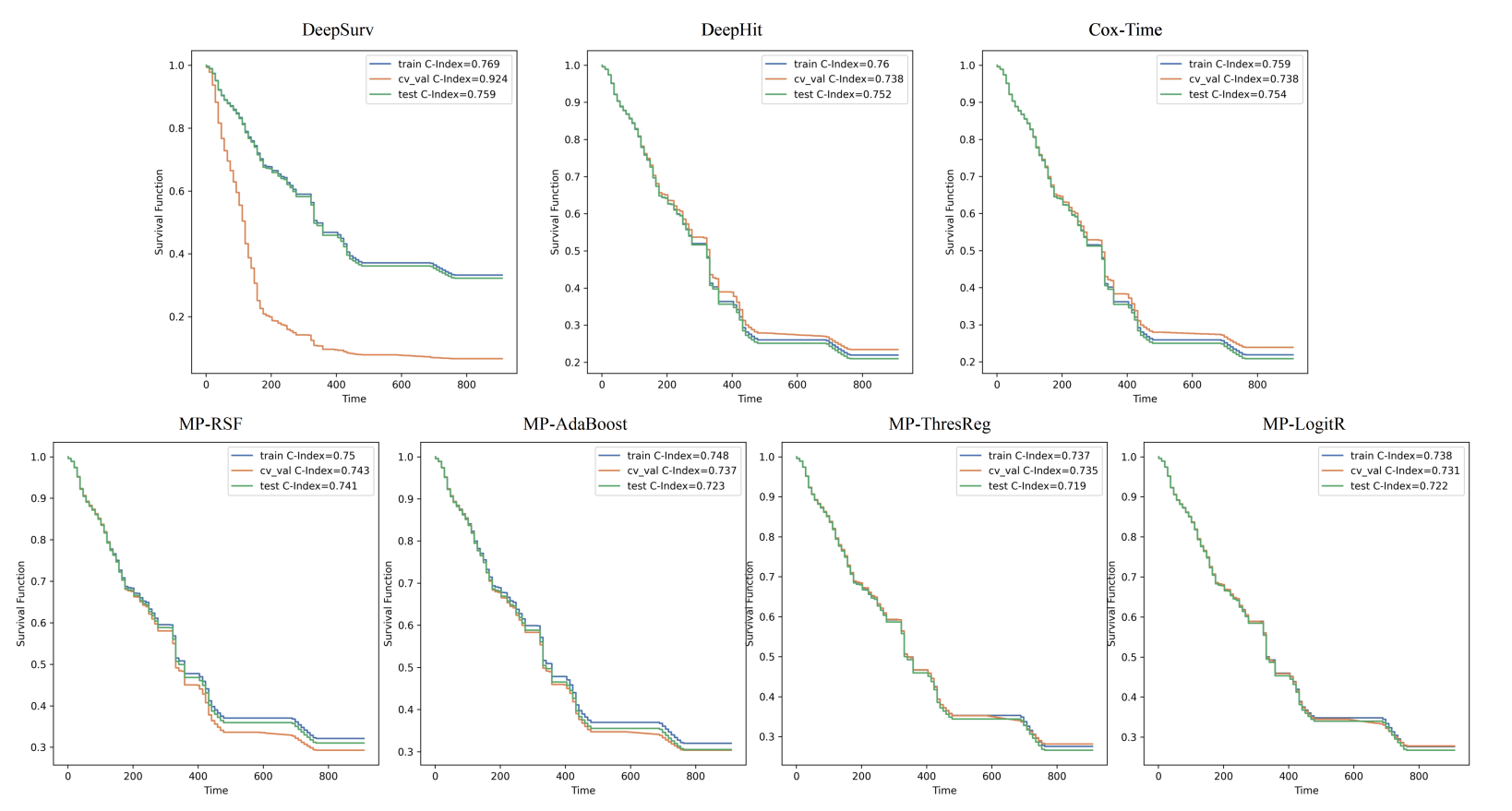
**Figure S1 Seven survival analysis models based on 16-parameter survival curves**

**References**

1. Bzdok, D., Altman, N., & Krzywinski, M. (2018). Statistics versus machine learning. Nature Methods, 15(4), 233-234.
2. Wang, K. L., Yap, E. S., Goto, S., et al. (2018). The diagnosis and treatment of venous thromboembolism in Asian patients. Thrombosis Journal, 16(1), 1-12.
3. Liu, S., Zhang, F., Xie, L., et al. (2019). Machine learning approaches for risk assessment of peripherally inserted central catheter-related vein thrombosis in hospitalized patients with cancer. International Journal of Medical Informatics, 129, 175-183.
4. Song, X., Lu, H., Chen, F., et al. (2020). A longitudinal observational retrospective study on risk factors and predictive model of PICC associated thrombosis in cancer patients. Scientific Reports, 10(1), 1-13.
5. Haider, H., Hoehn, B., Davis, S., et al. (2020). Effective ways to build and evaluate individual survival distributions. Journal of Machine Learning Research, 21(1), 3289-3351.
6. Lin, Y., Zeng, Z., Lin, R., et al. (2021). The Caprini thrombosis risk model predicts the risk of peripherally inserted central catheter-related upper extremity venous thrombosis in patients with cancer. Journal of Vascular Surgery: Venous and Lymphatic Disorders, 9(5), 1151-1158.
7. Hao, L., Kim, J., Kwon, S., et al. (2021). Deep learning-based survival analysis for high-dimensional survival data. Mathematics, 9(11), 1244.
8. Yue, J., Zhang, Y., Xu, F., et al. (2022). A clinical study of peripherally inserted central catheter-related venous thromboembolism in patients with hematological malignancies. Scientific Reports, 12(1), 9871.
9. Adeoye, J., Hui, L., Koohi-Moghadam, M., et al. (2022). Comparison of time-to-event machine learning models in predicting oral cavity cancer prognosis. International Journal of Medical Informatics, 157, 104635.
